# Exploring the Diversity of *Arcobacter butzleri* from Cattle in the UK Using MLST and Whole Genome Sequencing

**DOI:** 10.1371/journal.pone.0055240

**Published:** 2013-02-06

**Authors:** J. Yvette Merga, Nicola J. Williams, William G. Miller, Andrew J. H. Leatherbarrow, Malcolm Bennett, Neil Hall, Kevin E. Ashelford, Craig Winstanley

**Affiliations:** 1 Institute of Infection and Global Health, University of Liverpool, Liverpool, United Kingdom; 2 Produce Safety and Microbiology Research Unit, U. S. Department of Agriculture, Albany, California, United States of America; 3 Advanced Genomics Facility, Institute of Integrative Biology, University of Liverpool, Liverpool, United Kingdom; University of Vienna, Austria

## Abstract

*Arcobacter butzleri* is considered to be an emerging human foodborne pathogen. The completion of an *A. butzleri* genome sequence along with microarray analysis of 13 isolates in 2007 revealed a surprising amount of diversity amongst *A. butzleri* isolates from humans, animals and food. In order to further investigate *Arcobacter* diversity, 792 faecal samples were collected from cattle on beef and dairy farms in the North West of England. *Arcobacter* was isolated from 42.5% of the samples and the diversity of the isolates was investigated using multilocus sequence typing. An *A. butzleri* whole genome sequence, obtained by 454 shotgun sequencing of an isolate from a clinically-healthy dairy cow, showed a number of differences when compared to the genome of a human-derived *A. butzleri* isolate. PCR-based prevalence assays for variable genes suggested some tentative evidence for source-related distributions. We also found evidence for phenotypic differences relating to growth capabilities between our representative human and cattle isolates. Our genotypic and phenotypic observations suggest that some level of niche adaptation may have occurred in *A. butzleri*.

## Introduction

*Arcobacter* spp. are Gram-negative, spiral-shaped bacteria belonging to the family *Campylobacteraceae* and are ubiquitous in animals and the environment. *Arcobacter* spp. are considered to be emerging human foodborne pathogens, causing symptoms similar to campylobacteriosis, namely: nausea, persistent watery diarrhoea and severe stomach cramps [1], [2]. In addition, they were found to be the fourth most common *Campylobacteraceae* in studies of diarrhoeic human faecal samples in France [3] and Belgium [2].

*Arcobacter* spp. are associated with cattle, amongst other animals, having been isolated from beef and beef products [4], [5], [6], [7], [8], [9] and from the faeces of cattle in several studies worldwide [4], [5], [7], [8], [9], [10], [11], [12], [13], suggesting that cattle could act as a possible reservoir for transfer of the organism to humans. Little published data currently exist on the molecular epidemiology of *Arcobacter* in cattle in the UK [14].

In the study of any potential human foodborne pathogen it is important to understand both the disease-causing mechanisms involved in its pathogenicity, and the routes of transmission. An important step in gaining such knowledge is the identification of genotypic or phenotypic differences between, for example, pathogenic and non-pathogenic strains, or between strains associated with particular niches. Obtaining such data has become more feasible with the advent of whole genome sequencing, particularly with the emergence of high-throughput sequencing methods [15], and through typing methods such as multilocus sequence typing (MLST). Although *A. butzleri* genome sequences have been obtained from a human clinical isolate [16] and from isolates from microbial fuel cells [17], no cattle-associated isolates have been sequenced.

This study aimed to determine the level of diversity amongst *A*.*butzleri* isolates from cattle in the North West of England using MLST, and to further investigate the nature of this diversity using next-generation whole genome sequencing and phenotypic characterisation to compare an *A. butzleri* isolate from cattle with a human clinical isolate.

## Methods

### Ethics Statement

This work did not involve human or animal participants. Cattle faecal samples were collected from the ground after being observed being voided by the animals, and no direct contact with the animals was involved. Verbal consent was obtained from all farmers that participated in this study before sampling commenced, and feedback from the study was provided to all farmers after its completion.

### Sample Collection

Freshly voided faecal samples (n = 792) were collected from various management groups (calves and dry and lactating adults on dairy farms, fattening bulls, young stock, calves and heifers on beef farms) on two beef farms (Farms 1 and 4) and two dairy farms (Farms 2 and 3) in the North West of England, an area with a high level of dairy and some beef production. Each farm was visited five times during a 12-month period, from November 2007 to October 2008, with approximately eight weeks between each visit. Up to 50 samples were collected at each visit, using sterile plastic containers with a sterile plastic scoop to take approximately three grams of material from the centre of the freshly voided faeces. Samples were collected only immediately after the researcher had observed the faeces being voided, to avoid multiple samples being taken from the same animal, and to minimise contamination of the sample from the environment. Samples were transported to the laboratory at ambient temperature and processed within two hours.

### *Arcobacter* Isolation and Identification

One gram of each faecal sample was placed into nine ml of *Arcobacter* enrichment broth (18 g peptone, 1 g yeast extract, 5 g NaCl, 1L water) supplemented with five antibiotics (cefoperazone (16 mg), amphotericin B (10 mg), trimethoprim (64 mg), novobiocin (32 mg) and 5-fluorouracil (100 mg), Sigma-Aldrich, UK), [18]. The inoculated broth was incubated at 30°C under aerobic conditions for 24 hours. One loopful (approximately 10 µl) of broth from each sample was then streaked onto mCCDA agar with added CAT (cefoperazone, amphotericin, teicoplanin) supplement (Oxoid, Basingstoke, UK), [19] and incubated at 30°C under aerobic conditions for 48 hours. This method was chosen after a comparison of different methods for the isolation of *Arcobacter* spp. from cattle faeces [14]. Up to four colonies were selected from each plate based on morphology and Gram staining and purified on Columbia agar base (LabM Ltd. Bury, Lancashire) with 5% (v/v) defibrinated horse blood (Oxoid Ltd. Basingstoke, Hampshire) for 48 hours under aerobic conditions. Four colonies were selected for each isolate, as taking more was not feasible within this study. DNA was extracted from isolates using the Chelex-100 method [20] and isolates were identified to the species level using the PCR assays of Houf *et al*. [21] and Gonzalez *et al*. [22]. All isolates were also tested using the *Campylobacter*-specific PCR assay of Wang *et al*. [23] to further confirm that all isolates belonged to the genus *Arcobacter*; any *Campylobacter* isolates were discarded. Cultures were stored at −70°C using Cryovials (Prolab, Neston, UK).

### MLST

MLST was carried out according to the method of Miller *et al*. [24] on an Applied Biosystems 3130×l Genetic Analyser with ABI 3130 Data Collection software (v3.0), and using the FastSeq50 program.

Resulting sequence data were quality checked using the STARS software [25] with a connection to the *Arcobacter* pubMLST database (http://pubmlst.org/Arcobacter/), which allowed immediate identification of existing alleles. Any new alleles (i.e. those not recognised by the pubMLST system) were quality checked using CHROMAS (Technelysium, Australia) and submitted to the database curator to be assigned new allele numbers. Neighbour-joining trees were constructed from allelic sequence data at each locus using MEGA v4.1 [26].

The evolutionary relationships between sequence types were investigated by concatenating sequence data in the order *aspA*, *atpA*, *glnA*, *gltA*, *glyA*, *pgm* and *tkt* and constructing a neighbour-joining tree using MEGA v4.1 for complete allelic profiles. The evolutionary history was inferred using the neighbour-joining method. The evolutionary distances were computed using the Maximum Composite Likelihood method and codon positions included were 1st+2nd+3rd+noncoding. All positions containing gaps and missing data were eliminated from the dataset (Complete deletion option). Additional analysis was carried out using eBURST v3 (www.eburst.mlst.net).

### 454 Whole Genome Sequencing

*A. butzleri* strain “7h1h” was selected arbitrarily for sequencing from a collection of *A. butzleri* isolates obtained during a previous typing study of *Arcobacter* from cattle in the North West of England [14]. The strain was recovered from frozen culture on Columbia agar with 5% (v/v) defibrinated horse blood at 30°C for 72 hours. High quality DNA was extracted using a QIAamp mini DNA extraction kit (Qiagen, UK), and 454 pyrosequencing was performed using the 454 genome sequencer FLX, following the manufacturer’s instructions (Roche 454 Life Sciences, Basel, Switzerland).

The previously sequenced and fully annotated *A. butzleri* genome, RM4018 [16] was used for alignment and comparison. RM4018 is a derivative of the *A. butzleri* type strain, ATCC 49616.

The resulting shotgun reads were assembled to form a “pseudogenome” using the 454’s Newbler assembler software (Version 2.0.01.12, 454 Life Sciences), and using the published RM4018 genome for alignment [16] and comparison. GLIMMER version 3.02 (http://www.cbcb.umd.edu/software/glimmer/) was used to identify putative open reading frames (ORFs) and an in-house PERL script was used to identify and merge ORFs that were likely to have been split during the sequencing process. The resulting pseudogenome was annotated using ARTEMIS [27] and ACT; ARTEMIS Comparison Tool [28] software to align the 7h1h pseudogenome with the previously published RM4018 genome [16]. Each individual ORF, gene or coding region was separately investigated and ultimately assigned a putative name and function using the BLASTN online tool (http://blast.ncbi.nlm.nih.gov/Blast.cgi). No attempt was made to close gaps due to time and financial constraints, and because the pseudogenome obtained was suitable for a broad analysis with gaps present.

The entire 7h1h pseudogenome, along with the genome of RM4018, was uploaded onto the curated online SEED database [29], which features annotation and comparison facilities using “Rapid Annotation using Subsystems Technology”, RAST [30]. Using this software, the 7h1h pseudogenome was automatically annotated and its subsystems compared with those of RM4018. This whole genome shotgun project has been deposited at DDBJ/EMBL/GenBank under the accession number AFNL00000000. The version described in this paper is the first version, AFNL01000000.

### Phenotypic Characterisation of *A. butzleri* Isolates

Phenotypic characteristics of the two *A. butzleri* strains, 7h1h and RM4018, were compared using the Biolog Omnilog system (Biolog, CA, USA). Omnilog plate PM05 was first used to assess the growth of the two *Arcobacter* strains, using sodium succinate/ferric citrate as a carbon source. After both strains were grown successfully, plates PM02 (to test usage of carbon sources), PM03 (to test usage of nitrogen sources) and PM05 were then used for tests over a period of 96 hours, in order to monitor the growth in different conditions over a longer period. Finally, phenotypic testing using plates PM01-PM10 (for details see http://www.biolog.com/pmMicrobialCells.shtml) was carried out, using sodium succinate/ferric citrate as a carbon source, for the standard time of 72 hours in aerobic conditions. All tests were carried out in duplicate.

### Distribution of Variable genes Amongst *Arcobacter* Isolates

Based on the genome sequences of *A. butzleri* isolates 7h1h and RM4018, several variable regions were identified. In order to evaluate the distribution of these variable regions, seven pairs of oligonucleotide primers were designed using BatchPrimer3 (http://probes.pw.usda.gov/cgi-bin/batchprimer3/batchprimer3.cgi), to detect the presence of variable genes representing these regions in a panel of 50 *A. butzleri* isolates from different sources (Table 1). The isolates used in the screening panel were selected from available culture collections at the University of Liverpool and the Produce Safety and Microbiology Research Unit, USDA, Albany, California. Genes selected for investigation included genes that were absent from 7h1h but present in RM4018 and genes that were absent from RM4018 but present in 7h1h. The seven areas for investigation were selected because they represented areas of divergence between the two genomes which were not flanked by gaps in the sequence (in the case of 7h1h), and were not considered to be particularly variable areas. They each had different functions, and were found in different areas of either the 7h1h or RM4018 genome, as shown in Table 2. If a primer was designed based on a sequence present in the 7h1h genome but absent from the genome of RM4018 (i.e. Regions 3, 4 and 7), then strain 7h1h was used as a positive control in the PCR assays and RM4018 was used as negative control, as well as a second negative control containing only water. If primers were designed based on a sequence from the genome of strain RM4018 that was absent from the genome of strain 7h1h (Regions 1, 2, 5 and 6), then strain RM4018 was used as the positive control and strain 7h1h was used as the negative control. Both strains 7h1h and RM4018 were tested using each primer pair for quality control, as part of the primer design process, prior to beginning the assays of other strains.

**Table 1:** Details of the 50 *Arcobacter butzleri* isolates used to screen for the distribution of variable regions by PCR and the distribution of the genes.

Isolate	Species	Source	Origin	Region 1	Region 2	Region 3	Region 4	Region 5	Region 6	Region 7
C93	*A. butzleri*	Rabbit	UK	+	+					
C95	*A. butzleri*	Rabbit	UK							
C100	*A. butzleri*	Badger	UK	+	+		+			
C340	*A. butzleri*	Cattle	UK	+						
C341	*A. butzleri*	Cattle	UK	+						
C503	*A. butzleri*	Cattle	UK						+	
C505	*A. butzleri*	Cattle	UK	+					+	
W30385	*A. butzleri*	Water	UK	+			+		+	+
W30386	*A. butzleri*	Water	UK	+				+	+	
W30391	*A. butzleri*	Water	UK	+	+		+			
W30397	*A. butzleri*	Water	UK	+						
W30400	*A. butzleri*	Water	UK	+						
W30411	*A. butzleri*	Water	UK	+						
W30423	*A. butzleri*	Water	UK	+	+				+	+
W30429	*A. butzleri*	Water	UK	+	+			+	+	+
W32903	*A. butzleri*	Water	UK	+			+		+	
W30469	*A. butzleri*	Water	UK	+			+		+	
W32908	*A. butzleri*	Water	UK	+						
W32888	*A. butzleri*	Water	UK	+	+				+	
W32892	*A. butzleri*	Water	UK	+			+		+	
W32885	*A. butzleri*	Water	UK	+						
W32867	*A. butzleri*	Water	UK	+	+			+		+
W32862	*A. butzleri*	Water	UK	+	+			+	+	+
W32875	*A. butzleri*	Water	UK	+	+		+		+	
W33195	*A. butzleri*	Water	UK		+		+			
W33204	*A. butzleri*	Water	UK	+						
W33225	*A. butzleri*	Water	UK	+						
W33229	*A. butzleri*	Water	UK	+			+	+	+	
W33232	*A. butzleri*	Water	UK	+	+				+	
W33104	*A. butzleri*	Water	UK	+			+	+	+	
W32994	*A. butzleri*	Water	UK	+						
P31166	*A. butzleri*	Sheep	UK	+				+	+	
P31853	*A. butzleri*	Cattle	UK		+					
P32209	*A. butzleri*	Sheep	UK							
RM5556	*A. butzleri*	Human	Canada	+	+				+	+
RM3790	*A. butzleri*	Human	South Africa	+						
RM4129	*A. butzleri*	Human	South Africa	+	+					
RM4463	*A. butzleri*	Human	USA		+					
RM5230	*A. butzleri*	Human	Denmark	+				+	+	
RM5519	*A. butzleri*	Human	USA	+	+			+	+	
RM5529	*A. butzleri*	Human	USA	+	+			+	+	
RM5534	*A. butzleri*	Human	USA	+					+	
RM5542	*A. butzleri*	Human	USA	+	+			+	+	+
RM5543	*A. butzleri*	Human	Thailand		+			+	+	
RM5549	*A. butzleri*	Human	Thailand					+	+	
RM5530	*A. butzleri*	Human	USA					+	+	
RM5533	*A. butzleri*	Human	USA		+			+	+	
NC12481	*A. butzleri*	Human	USA		+			+	+	
**7h1h**	*A. butzleri*	Cattle	UK			+	+			
**RM4018**	*A. butzleri*	Human	USA	+	+			+	+	+
**Total**				**38**	**22**	**1**	**11**	**17**	**28**	**8**

**Table 2:** Oligonucleotide sequences and expected product sizes for primers designed to detect the presence of variable genes between *Arcobacter* isolates.

Region	Primer names	Variable gene status	Sequence (5′ – 3′)	Product size (bp)
Region 1	Abu987FAbu987R	Deleted from 7h1h	GCAGGAACAAAACTGCCTTC CATCATTTTCTTTTGCCCAAT	703
Region 2	Abu1814FAbu1814R	Deleted from 7h1h	TGGATAGTGCATATGCTTTTATGA CATCACCAGTTCCAACACCA	678
Region 3	Orf2356FOrf2356R	Deleted from RM4018	TTAGCCCCTCATTCGCCTAT AACTCCATGCCACAATTGAA	600
Region 4	Orf1258FOrf1258R	Deleted from RM4018	TGGTGTTGCAAATCCAATCT GCCAATTTGGATCTATTGTCG	704
Region 5	GlsAFGlsAR	Deleted from 7h1h	TTCCAGCTCTTGCAAATGTAAA ACCGCTTTTTCCAGGAAGTC	695
Region 6	Abu1030FAbu1030R	Deleted from 7h1h	GGGCACCAAACAATGCTTAT AGCAAGTGTTGCTGTTGCAC	692
Region 7	Orf1448FOrf1448R	Deleted from RM4018	GGCTCAAAAGGATACAATCCA AAACCAATTCCTATCCCATCTTC	683

The primer sequences and product sizes are shown in Table 2. 25 µl PCR reactions contained 0.5 µl 20 mM dNTP mix, 2.5 µl 10× PCR buffer (Abgene, UK), 1.5 mM MgCl_2_, 1 U *Taq* polymerase (Abgene, UK) and 1 µl template DNA. Reactions for Region 1, Region 3, Region 4 and Region 6 were carried out with an annealing temperature of 59°C, whereas reactions for Region 2, Region 5 and Region 7 used annealing temperatures of 65°C.

## Results

### *Arcobacter* Strains Isolated

A total of 792 faecal samples were collected from adult beef and dairy cattle, fattening beef cattle, weaned calves and unweaned calves. Three hundred and thirty-seven samples (42.5%) contained at least one species of *Arcobacter*. Of the *Arcobacter*-positive samples 113 (34%) contained *A. butzleri*, 185 (55%) contained *A. skirrowii* and 165 (49%) contained *A. cryaerophilus*, according to the results produced by the PCR assay of Houf *et al*. [21]. Twenty-four samples (7%) contained both *A. butzleri* and *A. skirrowii*, 32 (9%) contained both *A. butzleri* and *A. cryaerophilus*, 78 (23%) contained both *A. skirrowii* and *A*. *cryaerophilus*, and nine (3%) contained all three species. Three samples contained isolates which tested positive in the *Arcobacter* genus-specific PCR assay [22] but did not produce amplicons in the multiplex PCR assay [21] and so were not identified at the species level. All three were isolated from samples which contained at least one other species of *Arcobacter*. The prevalence of *Arcobacter* spp. in the samples appeared to peak during the summer months (May – August), with a clear drop in prevalence during February (data not shown).

### MLST

Based on preliminary work (unpublished), during which MLST was attempted on all isolates obtained in this study, 104 isolates showed clear, strong bands and produced complete MLST profiles. Four hundred and ten isolates produced poor quality sequence traces, comprising 250 *A. skirrowii* and *A. cryaerophilus* isolates, plus 160 *A. butzleri* isolates. It is possible that at least some of these isolates actually belonged to alternative *Arcobcter* species, such as *A. mytili* for example, which has been shown to be incorrectly identified as *A. skirrowii* using the PCR assay used in this study [31]. However, time and financial constraints prevented further testing of these isolates, hence only 104 of the isolates were selected for analysis, all of which belonged to the species *A. butzleri*. These comprised 43 different sequence types (STs), of which 41 were novel at the time of this study. Table 3 shows the abundance of each ST along with the distribution of STs across the four farms after all five sampling sessions. The most common type was ST-18, which constituted eight isolates from samples on three different farms (Farm 1, 3 and 4) and was isolated on two separate farm visits (December 2007; Farm 3 and July/August 2008; Farms 1 and 4). The remaining STs were isolated between one and six times (Table 2). ST-18 was the only ST to be isolated on more than one farm (Farms 1, 3 and 4). Three STs were isolated on multiple occasions on a single farm: ST-302 was isolated from Farm 1 during both May 2008 and July/August 2008, ST-308 was isolated from Farm 4 during both February 2008 and October 2008, and ST-346 was isolated from Farm 1 during May 2008 and July/August 2008.

**Table 3:** The distribution and abundance of the 43 sequence types.

ST	Number of times present (abundance)	Present in management group^farm number, sample time^
18	8	Lactating dairy cow^3, November 2007^Fattening bull^1, July 2008^Young beef stock^1,4, July 2008^
138	2	Fattening bull^4, February 2008^
293	4	Fattening bull^4, February 2008^
294	3	Beef calf^1, May 2008^Fattening bull^1, July 2008^
296	5	Fattening bull^1, February 2008^
298	3	Lactating dairy cow^3, February 2008^
301	6	Fattening bull^1, July 2008^Young beef stock^1, July 2008^
302	5	Beef calf^1, May 2008^Fattening bull^1, July 2008^
303	1	Fattening bull^1, July 2008^
304	1	Lactating dairy cow^2, November 2007^
306	4	Young beef stock^4, October 2008^
308	3	Young beef stock^4, October 2008^Fattening bull^4, October 2008^
309	3	Lactating dairy cow^2, November 2007^
310	2	Lactating dairy cow^2, November 2007^
311	1	Fattening bull^1, July 2008^
327	1	Fattening bull^4, July 2008^
328	1	Fattening bull^4, October 2008^
329	1	Fattening bull^4, July 2008^
330	2	Fattening bull^4, October 2008^
331	4	Fattening bull^4, October 2008^
332	2	Fattening bull^4, October 2008^
333	1	Young beef stock^1, October 2008^
335	3	Fattening bull^4, October 2008^
336	1	Young beef stock^4, July 2008^
337	1	Fattening bull^4, July 2008^
338	1	Fattening bull^4, October 2008^
339	3	Fattening bull^4, October 2008^
340	1	Young beef stock^1, July 2008^
341	2	Lactating dairy cow^3, October2008^
342	1	Beef calf^4, July 2008^
343	4	Beef calf^1, May 2008^
344	1	Fattening bull^4, July 2008^
345	1	Beef calf^1, July 2008^
346	5	Beef heifer^1, May 2008^Beef calf^1, July 2008^
348	1	Young beef stock^4, July 2008^
349	1	Fattening bull^4, October 2008^
350	1	Fattening bull^4, October 2008^
351	2	Fattening bull^4, October 2008^
352	3	Young beef stock^1, July 2008^
353	1	Young beef stock^1, May 2008^
354	4	Lactating dairy cow^2, October 2008^
356	1	Fattening bull^4, July 2008^
357	3	Fattening bull^4, October 2008^

Phylogenetic analysis of the sequence types found in this study, using MEGA and eBURST, revealed a high level of diversity amongst the isolates, with few bootstrap values above 50, and no clusters being identified by eBURST (data not shown).

### *A. butzleri* 454 Whole Genome Sequencing

The genome sequence data for the sequenced isolate, 7h1h, was deposited at DDBJ/EMBL/GenBank under the accession AFNL00000000 in order to facilitate comparisons and closer analysis. The resulting pseudogenome comprised 2,219,198 bp on 75 contigs. A total of 2420 predicted ORFs were identified. Although the genome is not complete and the authors did not attempt to close gaps, the mean coverage depth was 12.25, and the length and number of ORFs identified were both very similar to those reported for the completed genome of *A. butzleri* RM4018 (2,341,251 bp and 2259 respectively). Based on 454 sequence data there was no evidence for the presence of plasmids.

After blastn (nucleotide sequence) and blastp (protein sequence) comparison with the genome of *A. butzleri* RM4018, using the ACT program [28], 473 ORFs differing between the two genomes were identified. Two hundred and ninety six ORFs were present in 7h1h but absent from RM4018, 108 ORFs were absent from 7h1h but present in RM4018 and 69 ORFs were divergent (more than 10% difference in protein sequence when compared to the nearest matching ORF in RM4018). Table S1 shows a list of the ORFs identified in 7h1h using the ACT program, along with the nearest blastp result for each. Of the 1676 “core” genes identified by Miller *et al*. [16], 97.73% were also present in strain 7h1h. Those that were absent are presented in Table S2.

Table S3 shows all regions of greater than 5 kb that were novel to 7h1h (regions that were not present in the genome of isolate RM4018), the majority of which feature outer membrane proteins as well as some flagellar and phage-related proteins.

RAST annotation and comparison of the two genomes revealed 363 genes that differed between 7h1h and RM4018 (139 fewer than were identified using ACT). These comprised several sensing and survival-related genes, including genes encoding five TonB-dependent receptors, seven methyl-accepting chemotaxis proteins (five of which were present in 7h1h only) and five two-component sensing systems (three of which were present in 7h1h only). Table S4 shows a list of differences between 7h1h and RM4018 predicted by the RAST system.

Analysis of the *A. butzleri* 7h1h pseudogenome subsystems (defined in this case as groups of genes relating to a particular function), using RAST revealed 16 putative subsystems in the category “virulence, disease and defence”, all of which were related to antibiotic resistance. These comprised two subsystems relating to copper homeostasis, seven relating to cobalt, zinc and cadmium resistance, two relating to fluoroquinolone resistance, three relating to arsenic resistance, one relating to copper tolerance and homeostasis and one relating to a beta-lactamase. No subsystems relating to known adhesion, toxin production or other virulence-related factors were identified. Comparatively, RAST analysis of the genome of isolate RM4018 revealed 35 subsystems relating to “virulence disease and defence”, all of which were again related to antibiotic resistance. RAST also identified no genes relating to adhesion, toxin production or other virulence-related factors. Table 4 lists the different numbers of subsystems in each category identified by RAST in both isolates 7h1h and RM4018.

**Table 4:** Subsystems identified by RAST in isolates 7h1h and RM4018.

Subsystem Feature	Number in 7h1h Genome	Number in RM4018 Genome
Cofactors, Vitamins, Prosthetic Groups, Pigments	68	144
Cell Wall and Capsule	54	79
Virulence, Disease and Defence	16	35
Adhesion	0	0
toxins and superantigens	0	0
bacteriocins and ribosomally synthesized antibacterial peptides	0	0
resistance to antibiotics and toxic compounds	16	35
virulence, disease and defence – no subcategory	0	0
Detection	0	0
invasion and intracellular resistance	0	0
Potassium Metabolism	15	18
Photosynthesis	0	0
Miscellaneous	5	67
Phages, Prophages, Transposable Elements, Plasmids	5	5
phage family-specific subsystems	0	0
phages, prophages, transposable elements	0	0
transposable elements	5	0
pathogenicity islands	0	4
gene transfer agent	0	0
plasmid related functions	0	0
phages, prophages	0	1
Membrane Transport	20	37
Iron Acquisition and Metabolism	1	0
RNA Metabolism	30	68
Nucleosides and Nucleotides	26	43
Protein Metabolism	69	174
Cell Division and Cell Cycle	13	21
Motility and Chemotaxis	12	60
Regulation and Cell Signalling	8	14
Secondary Metabolism	0	5
DNA Metabolism	46	64
Fatty Acids, Lipids and Isoprenoids	48	33
Nitrogen Metabolism	16	12
Dormancy and Sporulation	0	1
Respiration	49	71
Stress Response	39	58
Osmotic stress	0	1
Dessication stress	0	0
Oxidative stress	21	29
Cold shock	1	1
Heat shock	14	13
No subcategory	0	12
Detoxification	3	3
Periplasmic stress	2	2
Metabolism of Aromatic Compounds	1	7
Amino Acids and Derivatives	98	229
Sulfur Metabolism	11	11
Phosphorus Metabolism	3	21
Carbohydrates	45	107

Twelve subsystems in 7h1h were found relating to motility and chemotaxis, comprised of mainly methyl-accepting chemotaxis proteins and ABC transport systems. RAST analysis also revealed eight subsystems relating to regulation and cell signalling, including three relating to murein hydrolase regulation and cell death, two relating to cAMP signalling and three relating to stringent response and (p)ppGpp metabolism. In RM4018, RAST identified 60 subsystems relating to motility and chemotaxis. It is worth noting, however, that these results are based on an incomplete genome, and after closing gaps in the 7h1h pseudogenome some differences may prove to be redundant.

Miller *et al.* [16], after comparing isolate RM4018 with 12 additional *A. butzleri* strains, produced a list of 42 genes that were present in RM4018 only. Three of these genes were also present in isolate 7h1h. Flagellar P-ring protein Flgl (AB0198) was present with 98% homology, DEAD/DEAH box helicase domain protein AB1337 was present with 81% homology and hypothetical protein AB1338 was present with 87% homology.

### Phenotypic Comparison of *A. butzleri* Isolates

**Figure 1:**
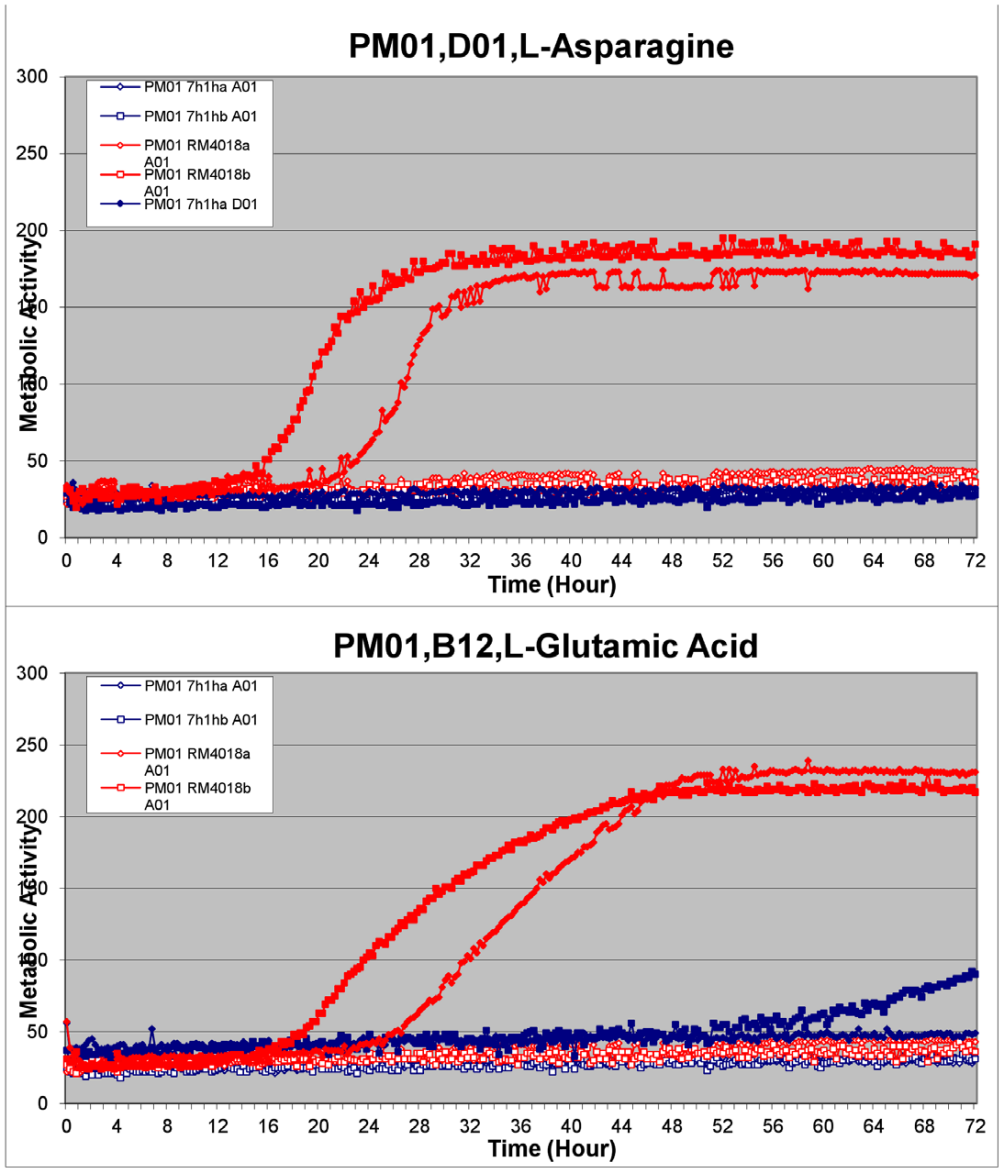
Example of metabolic variation between sequenced isolates. Omnilog output chart showing the growth curves of 7h1h (blue) and RM4018 (red) using L-asparagine and L-glutamic acid as carbon sources over a time period of 72 hours. Each isolate was tested in duplicate, hence 7h1ha, 7h1hb, RM4018a and RM4018b. The red and blue lines along the bottom of the charts include negative controls for each isolate.

Phenotypic comparisons using Omnilog analysis of the two *A. butzleri* strains, 7h1h and RM4018, revealed a number of phenotypic differences. The main results of note are presented in Table 5. In terms of metabolic differences, RM4018 utilised certain carbon and nitrogen sources more effectively than 7h1h, whereas 7h1h was able to utilise some sulphur and phosphorus sources that RM4018 did not. Figure 1 shows an example of two growth curves produced using the Omnilog system, indicating more abundant growth by strain RM4018 using L-asparagine and glutamic acid as carbon sources.

**Table 5:** The main phenotypic differences as determined by Omnilog analysis of 7h1h and RM4018.+represents growth and – represents no growth of the isolate on the omnilog plate.

Omnilog plate name	Type of test	Growth on substance	Result RM4018	Result 7h1h
PM01	Carbon sources	L-asparagine	+	–
PM01	Carbon sources	L-glutamic acid	+	–
PM03	Nitrogen sources	L-glutamine	+	–
PM03	Nitrogen sources	L-tryptophan	+	–
PM03	Nitrogen sources	L-tyrosine	+	–
PM04	Phosphorus and sulphur sources	Taurine	–	+
PM04	Phosphorus and sulphur sources	Butane sulfonic acid	–	+
PM04	Phosphorus and sulphur sources	Methane sulfonic acid	–	+
PM04	Phosphorus and sulphur sources	L-cysteic acid	–	+

### Distribution Of Variable Genes amongst *Arcobacter* Isolates

In the study of Miller *et al*. [24], 13 strains of *A. butzleri* from human and other sources were subjected to microarray analysis. Comparative genomic indexing of these strains revealed 1676 core genes, which were present in all 13 strains. Thirty-eight of these were not found in the incomplete 7h1h genome.

After annotation of the *A. butzleri* 7h1h genome sequence, seven predicted genes, including four that were absent from 7h1h when compared to RM4018, and three genes (Region 1; AB0987, Region 2; AB1814 and Region 6: AB1027) previously identified as core genes by Miller *et al*. [22] which were absent from 7h1h, were selected for the PCR screening of a panel of fifty *A. butzleri* isolates from different sources. Isolates 7h1h and RM4018 were included in the PCR assays and the results for these isolates in Table 1 are based on the PCR results. Table 1 shows the results of the assays, indicating whether isolates were PCR-positive or PCR-negative. Variable distribution of the selected regions was found amongst the isolates screened by PCR.

## Discussion

An overall prevalence of *Arcobacter* spp. of 42.5% was found in 792 cattle faecal samples. This figure is similar to other studies of *Arcobacter* spp. in cattle in Europe, which found the overall prevalence of *Arcobacter* spp. in cattle to be 27.3% [32], 39% [11] or 41.7% [33]. A previous study of *Arcobacter* spp. in cattle in the North West of England found *A. butzleri, A. skirrowii* and *A. cryaerophilus* amongst animals in the same geographical area [14], with a prevalence ranging from 84.5% (in weaned dairy calves) to 48.9% (dry adult dairy cows; unpublished data). However, it should be noted that although the species-specific PCR assay used in this study was designed to identify *A. butzleri*, *A. skirrowii* and *A. cryaerophilus* [21], it has been shown occasionally to misidentify other species. For example, *A. mytili* was incorrectly identified as *A. skirrowii* using this assay [31]. False negative results can also occur [14]. Since the aim of this study was to identify *A. butzleri*, *A. skirrowii* and *A. cryaerophilus*, we considered it suitable for use, but the possibility of some misidentification of isolates cannot be ignored.

Consistent with previous studies [14], [16], MLST analysis of a selection of *A. butzleri* isolates revealed a considerable amount of diversity between the isolates. Of 104 *A. butzleri* isolates typed here, 43 different sequence types were identified, the most common of which was present in just 8 isolates. Forty-one of the STs were novel at the time of this study. In a previous study of *Arcobacter* spp. from cattle in England, a similar level of diversity was determined, with 11 STs present in 39 *A. butzleri* isolates, all of which were novel at the time of study [14]. The fact that *A. skirrowii* and *A. cryaerophilus* isolates could not be accurately typed using MLST meant that only *A. butzleri* isolates were typed. As mentioned earlier, the PCR assay [21] is known to misidentify species on occasion. Although it is unlikely that all *A. skirrowii* and *A. cryaerophilus* species in this study were misidentified, the shortcomings of this assay may be responsible for the failure of MLST in a small number of cases. MLST primers used here were specifically designed to type *A. butzleri, A. skirrowii* and *A. cryaerophilus*, so the erroneous presence of some other species (e.g. *A. mytili*) amongst the *A. skirrowii* and *A. cryaerophilus* isolates might explain their failure to produce usable sequence traces in the MLST.

ST-18 was the only sequence type to be found on more than one farm (Farms 1, 4 and 3). Farms 1 and 4 were very close geographically (approximately 2 miles apart), while Farm 3 was slightly further away (approximately 15 miles from Farms 1 and 4). The presence of a shared ST suggests that the isolates may have had a common origin, for example a market or cattle breeder. A larger-scale study of cattle farms, markets, breeders and other possible sources of contamination in the area would be required to verify this. ST-18 was also found during different time periods (Farms 1 and 4 during July/August 2008 and on Farm 3 in December 2007). Other persistent isolates were ST-302 (found on Farm 1 in May and July/August 2008), ST-308 (found on Farm 4 during Feburary and October 2008) and ST-346 (found on Farm 1 during May and July/August 2008). Persistent STs such as these may either have survived within the herd for the duration of the gap between sampling sessions, or the herd may have become re-contaminated with the same ST, from the same original source. In the case of ST-308, it was detected on Farm 4 in February 2008 and later in October the same year, but was not detected at the two sampling sessions in between. However, it is possible that the presence of the ST continued throughout the year but was missed during sampling; fifty samples were taken at each sampling session, but this may not allow detection of every ST present in cattle on the farm, particularly as cattle populations on the farms ranged from 150 to over 400 individuals. Previous studies have also found high levels of diversity amongst *A. butzleri* isolates from both humans and animals using other typing methods such as macrorestricton pulsed-field gel electrophoresis, PFGE, [7], [34], [35] and ERIC-PCR [13], [32], [36], [37]. However, based on this study the reliability of ERIC-PCR as a specific typing method is questionable in light of the fact that binding sites for the ERIC-PCR primers were found to be absent from the two *A. butzleri* genome sequences, one of which was complete and closed.

It is possible that the very low annealing temperature of ERIC-PCR (25°C) [36], allows non-specific binding of the primers in *A. butzleri*, producing an almost random banding pattern for analysis. Short sequences corresponding to smaller sections of the ERIC-PCR primers can be found throughout the two genomes used in this study (data not shown), and it possible that these shorter sequences allow the primers to bind, resulting the bands observed in ERIC-PCR. If this is the case, then a higher annealing temperature (ideally 50°C or above) would prevent the ERIC primers from partially binding to shorter sections of DNA, thus making the technique more reliable. Previous studies have used ERIC-PCR and indicated good correlation with other typing methods [32], [37], suggesting that the method can be effective even in the absence of full ERIC sequences. In order to further explore the diversity highlighted by MLST, the whole genome sequence of a cattle-derived *A. butzleri* isolate, 7h1h, was obtained and annotated. Although gaps were not closed on the genome of *A. butzleri* 7h1h, there were some differences in comparison with the genome of *A. butzleri* RM4018, which had been isolated from a clinically-ill human and is a derivative of the *A. butzleri* type strain ATCC 49616 [16].

A precise representation of the variation between the two genomes is limited by the fact that the 7h1h genome has gaps, but a level of variation higher than might usually be expected in two isolates of the same species was apparent. However, it must be noted that upon closing gaps in the 7h1h sequence, the number of divergent genes could be significantly reduced.

Analysis using the RAST program revealed a substantial number of predicted differences between the two genomes which relate to sensing and survival. RAST predicts 7h1h to feature 5 novel Ton-B dependent receptors, 7 novel methyl-accepting chemotaxis proteins and 5 novel two-component sensing systems when compared to the genome of RM4018, all of which are related to environmental sensing and survival, and which have been implicated in niche adaptation [38]. Of course, it must be considered that the accuracy of this is limited somewhat by the presence of gaps in the 7h1h sequence.

It is interesting to note that a small number (n = 38; 2.27%) of the genes identified as “core genes” by microarray screening of 13 *A. butzleri* strains from different sources (excluding cattle) by Miller *et al*. [16] were absent from the incomplete genome of 7h1h. This may be related to the different sources of the strains used (the strains used for microarray screening were isolated originally from a variety of sources, except cattle), although it is likely that some of these “missing” genes may simply be due to gaps in the incomplete sequence. Screening of a larger number of strains from a wider variety of sources and geographical locations would demonstrate whether other cattle isolates show a similar divergence, and closing gaps in the 7h1h genome would provide a more accurate view.

The results obtained using the Biolog Omnilog phenotypic analysis highlighted some notable differences in the metabolic processes of the two *A. butzleri* isolates. *A. butzleri* strain RM4018 showed significantly faster growth than strain 7h1h when L-asparagine was used as a carbon source. L-asparaginases in Gram-negative bacteria have been linked to anaerobic fumarate respiration by providing aspartate, which is converted to fumarate. The inability of strain 7h1h to utilise L-asparagine suggests absence from the 7h1h genome of one or more genes relating to L-asparagine use, indicating that this amino acid may not be required for respiration in 7h1h. The genomes of 7h1h and RM4018 feature single predicted L-asparaginase genes which are highly similar (98% at the nucleotide level). However, the genome of *A. butzleri* 7h1h posesses only one putative glutamine-hydrolyzing asparagine synthetase, *asnB1,* whereas the genome of RM4018 posesses three: *asnB1* (glutamine-hydrolysing, 97% nucleotide similarity to that of *A. butzleri* strain 7h1h), *asnB2* and *asnB*. Fumarate is obtained from food, and the very different diets of cattle and humans undoubtedly results in different levels of fumarate being available in the guts of humans and cattle. The two strains of *A. butzleri* might have adapted according to the availability of fumarate in these very different environments, and the difference in the distribution of these genes could explain the difference in L-asparagine use by the two strains.

In addition, *A. butzleri* RM4018 was able to utilise L-glutamic acid as a carbon source, while *A. butzleri* 7h1h was not. The genome of *A. butzleri* 7h1h posesses two putative glutamine synthase genes and several putative glutamine amidotransferase genes, whereas the genome of *A. butzleri* RM4018 posesses the same genes with a high level of similarity, plus one additional gene, *glsA*, which encodes glutaminase A, the enzyme required to convert glutamine into glutamate. This may explain the phenotypic difference in glutamic acid metabolism shown by the phenotypic array as a similar result was found when using L-glutamine as a nitrogen source. Again, differences between the diets of humans and cattle lead to differences in the availability of glutamine in the gastrointestinal tract, creating two very different niches for *A. butzleri* habitation.

Different total numbers of divergent genes were obtained using the two programs; RAST and ACT. Both programs perform different functions (RAST identifies and compares functions and subsystems while ACT works using BLASTn or BLASTp alignment), and work using different algorithms. Due to the different nature of the two programs, it is inevitable that some differences in the results will occur. The fact that the 7h1h pseudogenome has gaps may also have affected the outcome from either program.Seven PCR assays were used to screen the distribution of variable regions that were either present in RM4018 as “core” genes [16] but absent from 7h1h or vice versa (Table 2). In the PCR assay, the ORF targeted in Region 1 in RM4018 was predicted to encode a transcriptional regulator of the *gntR* family that was present in the genome of strain RM4018 (AB0987) but absent from the genome of strain 7h1h. The *gntR* family of transcriptional regulators are responsible for transcription of a variety of different proteins and are widely distributed throughout the bacterial kingdom. It was identified as one of the “core” genes by [16] after microarray analysis of 13 *A. butzleri* isolates. In this study, 76% of the isolates screened were PCR-positive for this gene, supporting its status as a “core” gene.

The ORF representing Region 2 encodes a putative O-antigen polymerase in RM4018 (AB1814) and was deleted from the genome of 7h1h. The O-antigen is a polysaccharide found on the outer surface of bacterial cells, and is linked to virulence and host immune response. Overall 44% of isolates tested were positive for Region 2, with 60% of human isolates, 60% of wildlife isolates (isolates from one badger and two rabbits), and 36% of water isolates carrying this ORF. It was present in 16% of cattle-derived isolates, a lower frequency of distribution when compared to the other groups.

Region 3 encodes a putative glycerol phosphotransferase gene in the genome of 7h1h, which was deleted from RM4018. The only isolate PCR-positive for this ORF was 7h1h, suggesting that this enzyme is not essential for the survival of *A. butzleri* overall.

Region 4 encodes a putative toxin secretion ABC transporter in 7h1h which was identified as “divergent” in the comparison of the two genomes (43% similarity to a toxin secretion ABC transporter (ATP-binding and membrane protein) in RM4018). This region was present in 37.5% of water isolates and in one of the three wildlife isolates tested. It was absent from all human and sheep isolates, and 7h1h was the only cattle-derived isolate to test PCR-positive for this ORF.

Region 5 encoded the gene *GlsA*, and was deleted from 7h1h in comparison to RM4018. This gene was common in human-derived isolates (67%) and present in 29% of water-derived isolates, but absent from all cattle and wildlife isolates. Region six was found frequently in cattle, water and human isolate groups, but was not present in all isolates and Region 7 (present in 7h1h but deleted from RM4018) was uncommon overall, being present in just 16% of isolates. None of the seven genes were present in the single sheep-derived isolate tested. Further study of sheep-derived *Arcobacter* isolates would be required to determine whether this reflects significant diversity amongst *Arcobacters* from sheep.

Differences in the carriage of these genes amongst *A. butzleri* isolates from different sources suggests some level of niche adaptation may occur in *A. butzleri*. However, our data rely on single PCR assays which may be confounded by minor sequence variations. A larger, more detailed study would be required to reveal significant niche-associated differences in the carriage of these genes and other genes of interest.

This study compared two *A. butzleri* isolates from very different niches. There are likely to be many environmental differences between the gut of a healthy dairy cow and a clinically ill human, to which adaptation may occur. As mentioned earlier, the very different diets of humans and dairy cattle will provide different levels of nutrients such as amino acids and other proteins utilised for bacterial growth. Physical conditions in a ruminant gut will naturally be very different to those in a human gut, possibly driving adaptation. In addition, changes occurring in the human gut during clinical illness such as diarrhoea may also differ greatly from conditions in a ruminant gut or in an external environment such as water, where *Arcobacter* spp. have been frequently found. It should also be considered that many strains colonising humans may have come from a poultry source, and may have adapted to this niche prior to being isolated from humans. Adaptation may occur to suit any of these environments, resulting in genetic differences between strains.

The results of this study suggest that a large amount of diversity exists within the *Arcobacter* genus in a cattle reservoir, which would be further demonstrated by additional typing studies, using techniques such as MLST on more isolates. Our observations provide tentative evidence that differences in survival and sensing systems may be related to the source of the isolate, and might play a role in niche specialisation. However, these assumptions are based on an incomplete genome and this must be kept in mind when interpreting this data. Targeted studies with larger numbers of additional isolates from multiple other sources would help to clarify any relationships.

With the advance of genome sequencing technologies, the publication of additional *A. butzleri* and other *Arcobacter* spp. whole genome sequences will allow better understanding of the ecology and potential pathogenicity of this organism, and can confirm whether niche adaptation does occur in *A. butzleri* populations. Further study of human clinical isolates is needed to further elucidate the relationships between human and animal-derived *Arcobacters* and isolates from other sources.

Closure of the gaps in the 7h1h pseudogenome will undoubtedly reveal the true levels of diversity between the two strains in this study, and allow comparison with additional published *A. butzleri* genomes.

## Supporting Information

Table S1 Table showing all ORFs identified in the 7h1h sequence using the ACT program, with BLASTp results for each.
(XLSX)

Table S2 Table showing those “Core” genes which were absent from the 7h1h sequence.
(DOCX)

Table S3 Table showing all regions of greater than 5kb which were novel to 7h1h.
(XLSX)

Table S4 A list of differences between 7h1h and RM4018 as predicted by the RAST program.
(XLSX)
