# Supplementary material for: Exploring the Diversity of Arcobacter butzleri from Cattle in the UK Using MLST and Whole Genome Sequencing
Source: PLoS One. 2013 Feb 6;8(2):e55240. doi: 10.1371/journal.pone.0055240 (PMC3566208; doi:10.1371/journal.pone.0055240)
Supplement: Table S2 — Table showing those “Core” genes which were absent from the 7h1h sequence. (DOCX) [file pone.0055240.s002.docx]

| **Name of Region on RM4018 Genome** | **Putative Function** |
| --- | --- |
| AB0225 | Conserved hypothetical DNA binding protein |
| AB0595 | Conserved hypothetical protein |
| AB0596 | Hypothetical TPR repeat protein |
| AB0597 | Transcriptional regulator, MarR family |
| AB0630 | Two-component response regulator |
| AB0631 | Two-component sensor histidine kinase |
| AB0632 | Methyl-accepting chemotaxis protein |
| AB0987 | Transcriptional regulator, GntR family |
| AB0988 | FMN-binding protein |
| AB0990 | AcrB/AcrD/AcrF family protein |
| AB0991 | AcrB/AcrD/AcrF family protein |
| AB0992 | AcrA/AcrE family protein |
| AB0993 | Outer membrane efflux protein |
| AB1026 | Two-component sensor histidine kinase |
| AB1027 | Two-component response regulator |
| AB1028 | Outer membrane efflux protein, putative |
| AB1029 | Conserved hypothetical protein |
| AB1030 | Hypothetical membrane protein |
| AB1031 | Putative membrane protein |
| AB1032 | Peptidase, M50 family |
| AB1136 | Conserved hypothetical protein |
| AB1385 | Hypothetical protein |
| AB1386 | Conserved hypothetical protein |
| AB1387 | Conserved hypothetical protein |
| AB1391 | Conserved hypothetical protein (DUF1706 domain protein) |
| AB1404 | McrBC endonuclease McrB, putative |
| AB1414 | Hypothetical protein |
| AB1415 | Conserved hypothetical protein |
| AB1430 | Sigma factor, ECF family |
| AB1804 | Conserved hypothetical membrane protein |
| AB1810 | Conserved hypothetical protein |
| AB1811 | Probable glycosyltransferase |
| AB1812 | Conserved hypothetical protein |
| AB1813 | Glycosyltransferase |
| AB1814 | Putative O-antigen polymerase |
| AB1815 | Aminotransferase, DegT/DnrJ/EryC1/StrS family |
| AB1817 | Glucose-1-phosphate thymidylyltransferase |
| AB1820 | Conserved hypothetical protein |
